# Neural networks and arbitrage in the VIX: A deep learning approach for the VIX

**DOI:** 10.1007/s42521-020-00026-y

**Published:** 2020-08-13

**Authors:** Joerg Osterrieder, Daniel Kucharczyk, Silas Rudolf, Daniel Wittwer

**Affiliations:** 1 School of Engineering, Zurich University of Applied Sciences, 8401 Winterthur, Switzerland; 2 Faculty of Pure and Applied Mathematics, Hugo Steinhaus Center, Wroclaw University of Science and Technology, 50-370 Wroclaw, Poland; 3 Nexoya Ltd., 8005 Zurich, Switzerland; 4 AGCO International GmbH, 8212 Neuhausen, Switzerland

**Keywords:** VIX, SPX, Neural network, LSTM, Deep learning, Arbitrage, Market manipulation, Random forests, A00, C00, G00

## Abstract

The Chicago Board Options Exchange Volatility Index (VIX) is considered by many market participants as a common measure of market risk and investors’ sentiment, representing the market’s expectation of the 30-day-ahead looking implied volatility obtained from real-time prices of options on the S&P 500 index. While smaller deviations between implied and realized volatility are a well-known stylized fact of financial markets, large, time-varying differences are also frequently observed throughout the day. Furthermore, substantial deviations between the VIX and its futures might lead to arbitrage opportunities on the VIX market. Arbitrage is hard to exploit as the potential strategy to exploit it requires buying several hundred, mostly illiquid, out-of-the-money (put and call) options on the S&P 500 index. This paper discusses a novel approach to predicting the VIX on an intraday scale by using just a subset of the most liquid options. To the best of the authors’ knowledge, this the first paper, that describes a new methodology on how to predict the VIX (to potentially exploit arbitrage opportunities using VIX futures) using most recently developed machine learning models to intraday data of S&P 500 options and the VIX. The presented results are supposed to shed more light on the underlying dynamics in the options markets, help other investors to better understand the market and support regulators to investigate market inefficiencies.

## Introduction

The VIX index has been subject to claims of manipulation over the last few years, see, e.g., Griffin and Shams (2017). We will analyze intra-day data for S&P 500 options to predict the VIX, and, using neural networks, to show how one can exploit potential arbitrage opportunities without having to buy and sell several hundred out-of-the-money put and call options, as described by the VIX methodology (Exchange 2009).

On February 5, 2018, the VIX moved the most in a single day in the index’s 25-year history. The VIX and the VIX futures deviated substantially from each other on that day, which was one of the motivations behind our analysis. Another anecdotal evidence, showing the impact of SPX option trades on the VIX, is April 18, 2018. Shortly after the monthly settlement auction that determines the price for VIX options and futures, the VIX spiked as much as eleven percent within 1 h. A trade of 13,923 May puts on the S&P 500 with a strike price of 1200, worth roughly $2.1 million, took place just as markets opened at 9:30 a.m. (Lu Wang and Kawa 2018)

The Chicago Board Options Exchange (CBOE) Volatility Index (VIX) is a mathematical formula which is considered the most important benchmark for implied volatility on the US stock market. Generally, the VIX sheds light on how investors “feel” about the market, hence its nickname, the “fear gauge.” Its design is such that it tries to approximate the 30-day implied volatility of at-the-money options on the S&P 500. Andersen et al. (2015) demonstrate that the VIX index can exhibit deviations from true volatility due to the inclusion of illiquid options. The methodology that is applied throughout the paper is based on the long short-term memory (LSTM) network architecture which is used for analysing the time-series of S&P 500 option quotes and predict the VIX. Artificial neural networks have seen a revival in the last few years, due to better mathematical techniques for backpropagation but also due to the enormous computing power that is nowadays available. Recurrent neural networks which are composed of LSTM units are simply referred to as an LSTM network in the following. LSTM was proposed in 1997 by Hochreiter and Schmidhuber (1997) and improved in 2000 by Felix Gers’ team (Gers et al. 2000).

Based on research by Kumar and Seppi (1992) and Spatt (2014), the S&P 500 options and the VIX are markets with features that might leave it open to manipulation: the SPX options market with illiquid instruments and high transaction costs facing a large and liquid VIX derivatives market.

Any mispricings in the VIX should be arbitraged away by trading the VIX. However, this is not directly possible. One has to fall back to using VIX futures as a proxy for the VIX, to use the S&P 500 options to construct the VIX or to find similarly suitable proxies, such as a limited set of put and call options to approximately compute the VIX. Our goal is to use a neural network to show how to predict the VIX over the next 60 s. The results are twofold: first, we show how one can train the neural network to predict the VIX, without knowing the actual theoretical formula, by simply using the same set of options as in the CBOE VIX methodology. Second, we can also train the network on the subset of options that are most relevant, those out-of-the-money options that are closest to the current forward of the S&P index. There is a substantial benefit when applying this approach. By just using a small subset of all options for the VIX calculation and knowing their weights, we can predict the VIX with high accuracy over the next four quoted time-intervals which are 60 s, beating the trivial approach of using the last observation as a prediction for the future value. As another application, knowing the weights for combining the current prices of liquid calls and puts to get a 60 s-ahead forecast of the VIX could be useful for approximately hedging a variance swap entered into 60 s later, since the square of the VIX can be seen as the fair strike of a variance swap.

The remainder of this paper is organized as follows: Sect. 2 provides a literature review of relevant studies that use deep learning to analyse financial data and an overview of academic literature related to the VIX. Section 3 describes the historical relevance of the VIX for financial markets and introduces artificial neural networks as the method of choice for our analysis. In Sect. 4 an in-depth description of the data is provided. Section 5 gives more background on the VIX and its relation to the options market by analysing the VIX formula, a prediction strategy and VIX futures. The design of the neural network, the implementation of the LSTM model as well as the results are described in Sect. 6. Finally, Sect. 7 discusses the impact of this research and potential future applications.

## Literature review

There have been many studies dedicated to investigating deep learning’s applicability to financial problems involving classification and prediction. Most of those are forecasts of stock market returns. Olson and Mossman (2003) attempt to predict 1-year-ahead stock returns for 2352 Canadian companies using 61 accounting ratios as input values and reported that neural networks outperform traditional regression techniques. Kryzanowski et al. (1993) found that neural networks correctly classify 72% of the returns to predict 1-year-ahead stock returns by using financial ratios and macroeconomic variables.

To predict 1-day-ahead stock returns for the S&P 500 constituents, Krauss et al. use deep neural networks, gradient-boosted trees and random forests. As a result, they show that combining the predictions of those three as an equal-weighted ensemble outperforms each individual model. Among each model, random forests outperform deep neural networks and gradient-boosted trees. Conversely, they stated that careful hyper-parameter optimization may still yield advantageous results for tuning intensive deep neural networks.

In 2016, Luca Di Persio and Oleksandr Honchar of the University of Verona completed a study that uses Artificial Neural Networks to predict stock market indices (Di Persio and Honchar 2016). They experimented with many different architectures using multi-layer perceptron, convolutional neural networks (CNN), and LSTM layers. Through a wavelet transformation (WT) technique, Periso and Oleksandr transformed their data before passing it through the CNN model, which produced the most accurate results out of all of the other models they used (including the CNN model without the transformed data). Another research team based in China similarly had success by combining WTs, stacked autoencoders (SAEs), and LSTM in a model for stock price forecasting (Bao et al. 2017). Both of these studies highlight the importance of transforming the data in some way before passing it through a deep learning model in order to decrease noise.

The paper by Hochreiter and Schmidhuber (1997) is a comprehensive source on LSTM networks. In this study, the authors explain the mathematics behind why LSTM networks are able to solve complex problems that other networks are not. They also experiment with different types of datasets and compare LSTM’s performance to other common networks. LSTMs and recurrent neural networks are still an area of intensive academic research and ongoing discussions. Recently, there has been a trend in handwritten text recognition with deep neural networks to replace 2D recurrent layers with 1D, and in some cases even completely remove the recurrent layers, relying on simple feed-forward convolutional only architectures. A more detailed discussion of that can be found in the 2018 paper of Moysset and Messina (2017). On the other hand, those two authors show that 2D-LSTM networks still seem to provide the highest performances. The most important work on manipulation in the VIX was written by Griffin and Shams (2017). They analyse market characteristics around the settlement of the VIX index in great details and show that volume spikes on S&P 500 index options at those times, but only for out-of-the-money options and more so for options with a higher and discontinuous influence on the VIX. Demeterfi et al. (1999) have done the first comprehensive analysis and derivation of the price of volatility and variance swaps. They explain the properties and the theory of both variance and volatility swaps. They show how a variance swap can be theoretically replicated by a hedged portfolio of standard options with suitably chosen strikes, as long as stock prices evolve without jumps. For volatility swaps they show that those can be replicated by dynamically trading the more straightforward variance swap. Andersen et al. (2015) demonstrate that the VIX Index has deviations from true volatility due to the inclusion of illiquid options. Futures and options on the VIX have a relatively large volume. The settlement value of those derivatives is calculated from a wide range of OTM put and call options with different exercise prices. A manipulator would have to influence exactly those prices of the lower-level OTM SPX options to influence the expiring upper-level VIX derivatives. The authors also show that fluctuations of illiquid OTM options lead to undesired variations of the VIX value. In 2017, Li (2017) shows that the CBOE VIX methodology underestimates implied variance in general. The under-estimation increases as the forward index value moves higher and away from a strike price, peaks at the next strike, and resets to zero when passing the strike. He points out that a significant under-estimation can show up in related VIX indices such as the CBOE VVIX (the VIX of VIX) where fewer strikes are quoted. In 2018, Pimbley and Phillips (Pimbley and Phillips 2018) point out several aspects which show that the CBOE Volatility Index is prone to inadvertent and deliberate errors. They indicate several measures that can be taken to improve the index’s accuracy and curtail its susceptibility to misuses and falsifications.

## The VIX and deep learning

Here, we give some background information on the VIX and the deep learning technology that we apply. Section 3.1 discusses the relationship between the VIX and the S&P 500 options, while the required background on neural networks, which is needed to understand the deep learning architecture, is provided in Sect. 3.2.

### The CBOE Volatility Index

In this paragraph, a short overview of the historical evolution of a volatility index on the US equity market is provided. Additionally, the current CBOE methodology for the computation of the VIX is explained. In the sequel, the term relevant options means those options that are used in the calculation of the VIX based on the CBOE VIX White paper (Exchange 2009). Published by the CBOE, this volatility index is calculated using a weighted sum of mid-quotes, on out-of-the-money put and call options of the S&P 500 with a maturity between 23 and 37 days (Exchange 2009). Typically the VIX ranges between 10 and 30 points, major economic events being the exceptions. It cannot be traded directly, but there are many derivatives on the index, including options and futures. While entering the VIX as the square-root of weighted averages of prices, the SPX options contain much more information than the index itself, leading naturally to the possibility that there are different volatility surfaces implying the same VIX. Conversely, the same implied volatility can be achieved by different weighting and averaging schemes of the option prices, a feature which we will exploit later when applying our deep learning methodology.

#### Historical evolution of the VIX index

In 1987, Brenner and Galai first introduced the Sigma Index in an academic paper (Brenner and Galai 1989): “Our volatility index, to be named Sigma Index, would be updated frequently and used as the underlying asset for futures and options... a volatility index would play the same role as the market index play for options and futures on the index”. In 1992, The American Stock Exchange announced a feasibility study for a volatility index, proposed as “Sigma Index”. “SI would be an underlying asset for futures and options that investors would use to hedge against the risk of volatility changes in the stock market.” On January 19, 1993, the Chicago Board Options Exchange introduced the VIX. Developed by Robert Whaley, it was designed to measure the 30 days implied volatility of at-the-money (ATM) S&P 100 (OEX) option prices (Whaley 1993). Ten years later, the CBOE, together with Goldman Sachs, developed further computational methodologies which involved changing the underlying OEX to the S&P 500 (SPX). In general, using SPX options with more than 23 days and less than 37 days to expiration ensures that the VIX will always reflect an interpolation of two points along the S&P 500 volatility term structure (Exchange 2009).

Up until now, this new VIX has been based on the S&P 500 registered Index (SPXSM), the core index for US equities and estimates expected volatility by averaging the weighted quotes of SPX put and call options over a wide range of strike prices. In 2004, the CBOE began to introduce futures and 2 years later, in 2006, presented its new product, VIX options.

In 2014, another improvement was made by including SPX weekly options (SPXW), expiring on Fridays, in the calculation. This inclusion intends to more precisely reflect the 30 days expected volatility of the S&P 500.

#### How the VIX market works

The VIX is being disseminated every 15 s from 2:15 a.m. to 8:15 a.m. and from 8:30 a.m. until 3:15 p.m. Central Standard Time (CST). The final settlement value for VIX futures and options is based on a Special Opening Quotation (SOQ) of the VIX Index calculated using opening prices of constituent SPX and SPX Weekly options that expire 30 days after the relevant VIX expiration date. For example, the final settlement value for VIX derivatives expiring on November 21, 2018, will be calculated using SPX options that expire 30 days later on December 21, 2018.^1^The opening prices for SPX options used in the SOQ are determined by an automatic auction mechanism on CBOE options, which matches locked or inverted buy and sell orders and quotes resting on the electronic order book at the opening of trading (Exchange 2018). Even though the SPXW options expire at 3:00 p.m., the calculation for the settlement value takes place at the same time as the SPX options (8.30 a.m.).

#### The CBOE VIX formula explained

CBOE uses the following formula for the calculation of the VIX (Exchange 2009):

1 $$\begin{aligned} \sigma ^{2}=\frac{2}{T}\sum \limits _{i=1}^{n}\frac{\Delta K_{i}}{K_{i}^{2}}e^{rT}Q(K_{i})-\frac{1}{T}\left( \frac{F}{K_{0}}-1\right) ^{2}, \end{aligned}$$

where T is the time to expiration, r the risk-free interest rate (based on US. Treasury yield curves for the expiration dates of relevant SPX options), *F* the forward price of the S&P 500 index, $K_{0}$ the first strike below the forward index level *F* and $K_{i}$ the strike price of the i^th^ OTM option. The quote $Q(K_{i})$ is the mid-point of the bid-ask prices of the option with strike $K_{i}$. More precisely, *T* is defined as follows:

2 $$\begin{aligned} T=\frac{(M_{\text {current day}}+ M_{\text {settlement day}}+ M_{\text {other days}})}{\text {minutes in a year}} \end{aligned}$$

where M$_{\text {current day}}$ denotes the minutes remaining until midnight of the current day, M$_{\text {settlement day}}$ are the minutes from midnight until 8:30 a.m. for standard SPX options and minutes from midnight until 3:00 p.m. for SPXW expirations, M$_{\text {other days}}$ are the total minutes in the days between the current day and the expiration day of the options.^2^*F* is defined as

$$\begin{aligned} F=\text {Strike Price}+e^{rT}\cdot (\text {Call Price}-\text {Put Price}) \end{aligned}$$

Here, it should be pointed out that all calculations of the VIX are computed for the near- and next-term options. The CBOE distinguishes near-term options with a remaining time between 23 and 30 days and next-term options with a remaining term between 31 and 37 days.

When selecting the OTM puts you work successively from $K_0$ to the lower strikes and exclude all options with a zero-bid. If two consecutive zero bids occur, all options with lower strikes are no longer considered. Knowing all these rules and parameters one can easily calculate $\sigma _{1}^{2}$ and $\sigma _{2}^{2}$, which are the near- and next-term components of the VIX. To obtain the VIX value one takes a weighted 30-day average of $\sigma _{1}^{2}$ and $\sigma _{2}^{2}$

3 $$\begin{aligned} \text {VIX}=100\cdot \sqrt{\left[ T_{1}\cdot \sigma _{1}^{2}\cdot \left( \frac{N_{T_{2}}-N_{30}}{N_{T_{2}}-N_{T_{1}}}\right) +T_{2}\cdot \sigma _{2}^{2}\cdot \left( \frac{N_{30}-N_{T_{1}}}{N_{T_{2}}-N_{T_{1}}}\right) \right] \cdot \frac{N_{365}}{N_{30}}}, \end{aligned}$$

where

- $T_{1}=$ Time to expiry (as a fraction of the total number of minutes in a year) of the near-term options,
- $T_{2}=$ Time to expiry (as a fraction of the total number of minutes in a year) of the next-term options,
- $N_{T_{1}}=$ number of minutes to settlement of the near-term options,
- $N_{T_{2}}=$ number of minutes to settlement of the next-term options,
- $N_{30}=$ number of minutes in 30 days (43, 200) and
- $N_{365}=$ number of minutes in a 365-day year (525, 600).

Demeterfi et al. (1999) show how the VIX formula can be derived based on a Brownian motion process for the underlying, using Black-Scholes assumptions, by using Itô’s Lemma and approximating an infinite number of option strikes by a finite sum. Then, using various Taylor approximations as well as appropriate integral approximations, one arrives at the final formula for the VIX.

### Our network architecture: a recurrent neural network

In the following, we will describe our network architecture and its components.

Our approach uses recurrent neural networks (RNNs) together with long short-term memory (LSTM) units, which are a class of artificial neural networks where connections between nodes form a directed graph along a sequence. This allows it to exhibit temporal dynamic behaviour for a time sequence.

The major challenges of deep learning methods arise from the task of choosing the “best” model architecture. Facing the lack of computing power to test all possible model structures on any given data set, it is crucial to rely on previous research, data set characteristics, and intuition to design a deep learning model. David Wolpert, Mathematician and Santa Fe Institute Professor, describes the machine learning “no free lunch theorem” as follows: “for any two learning algorithms A and B... there are just as many situations (appropriately weighted) in which algorithm A is superior to algorithm B as vice versa.” Wolpert and Macready (1997). It follows that there is no universal model structure or learning algorithm, meaning different model structures give more accurate results on different data sets and for different purposes. There is also no universal guide on how to design a model, so intuition and experience are imperative in model design. Further complexities arise from underfitting and overfitting problems and from the task of how to efficiently train a neural network.

Following the proposed structure in Géron (2017), we describe the most important aspects of our network. Those are Initialization, Activation function, Normalization, Regularization, Optimizer and the learning rate schedule.

*Initialization* For our LSTM, we need to initialize the weights for the linear transformation of the input, the weights for the recurrent state and the bias vector. For those, we use the Glorot/Xavier uniform initializer (Glorot and Bengio 2010b), the orthogonal initializer (the weight vectors associated with the neurons in each layer are supposed to be orthogonal to each other) and zeros, respectively. The Glorot initializer achieves a good compromise for our desired requirement that the signal flows properly in both directions: in the forward direction when making predictions, and in the reverse direction when back-propagating gradients.

**Fig. 1:**
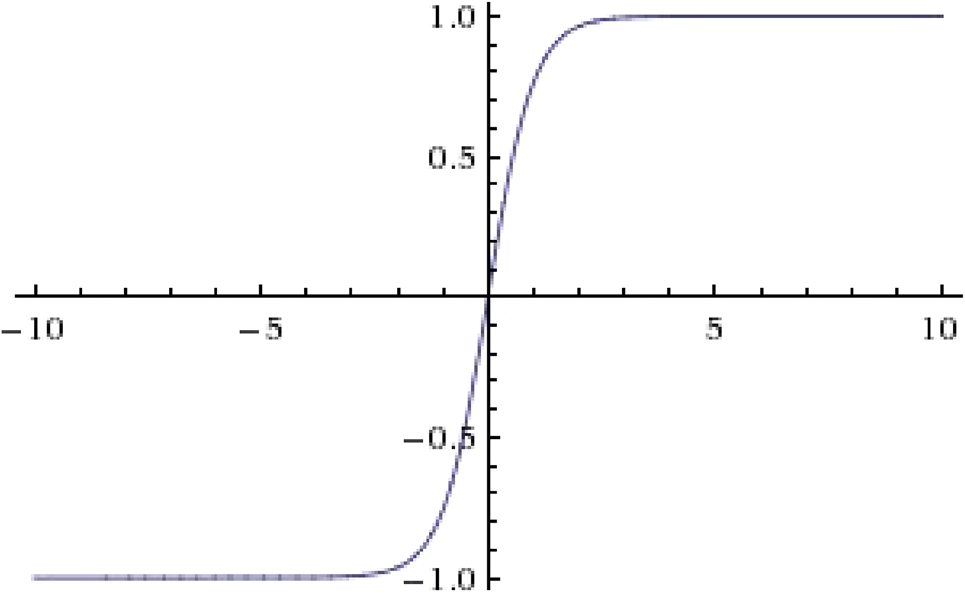
The Hyperbolic Tangent (tanh) activation function

*Activation function* Activation functions are used to introduce non-linearity to a network. In our case, the *tanh* activation function is used: The hyperbolic tangent (tanh):

4 $$\begin{aligned} \text {tanh}(x) = \frac{2}{1+e^{-2x}} -1 \end{aligned}$$

is a very popular and widely used activation function. It compresses the input to the range $(-\,1, 1)$ and provides a zero-centered output (Fig. 1).

*Normalization* In a 2015 paper, Ioffe and Szegedy (201) proposed a technique called Batch Normalization to address the vanishing/exploding gradients problems, and more generally the problem that the distribution of each layer’s inputs changes during training, as the parameters of the previous layers change. We have decided to not use batch normalization since we are only using a batch size of ten in our approach.

*Regularization* To reduce potential problems arising from overfitting, we use dropout at a rate of 0.1 as our regularization technique.

*Optimizer* As an optimizer, we are using the Adaptive Moment Estimation (ADAM), which is a method that computes adaptive learning rates for each parameter. In addition to storing an exponentially decaying average of past squared gradients, ADAM also keeps an exponentially decaying average of past gradients:

5 $$\begin{aligned} m_t= & {} \beta _1 m_t-1 + (1-\beta _1)g_t \end{aligned}$$

6 $$\begin{aligned} v_t= & {} \beta _2 v_t-1 + (1-\beta _2)g^2_t \end{aligned}$$

$m_t$ and $v_t$ are estimates of the mean and the uncentered variance of the gradients. $g_t$ denotes the gradient, i.e. the vector of partial derivatives of $f_t$ evaluated at timestep t. $\beta _1$ and $\beta _2$ are hyper-parameters that control the exponential decay rates.

Since $m_t$ and $v_t$ are initialized as vectors of 0’s they are biased towards zero. To counteract these biases, bias-corrected estimates are computed and used to update the parameters $\theta$ with the following ADAM update rule:

7 $$\begin{aligned} \theta _{t+1}=\theta _t - \frac{\eta }{\sqrt{\hat{v_t}}+\epsilon }\hat{m}_t \end{aligned}$$

Summarizing, the benefits of ADAM consist of an adaptive learning rate and momentum for each parameter, as well as a non-diminishing learning rate. On the downside, it does not have the ability to “look ahead” before taking the next step like other optimizers, which include an approximation of $\theta _{t+1}$ in the calculation.

*Learning rate schedule* The Adam optimizer is an adaptive learning rate algorithm, therefore, we just need to decide on an initial learning rate. For the momentum decay hyper-parameter, we use 0.9 and for the scaling decay hyperparameter, we use 0.999.

## Intraday SPX options and VIX spot data

Data is obtained directly from CBOE.^3^ The datasets contain SPX options as well as the VIX spot index, VIX futures and options on the VIX. We have intraday data for all data sets, for the S&P 500 options we have a 1-min granularity, the VIX itself is disseminated every 15 s and for VIX futures and options, we have a 1-s granularity. The period of examination is the 2-month period from January 2, 2018 until February 28, 2018, with the daily data available on trading days between 8:31 a.m. CST until 3:15 p.m. CST.

For the VIX index, only a particular subset of SPX options is used [see VIX white paper (Exchange 2009). Following this methodology, we remove all options, at a given point in time, that do not satisfy all of the following criteria:

- Expiration date between 23 and 37 days in the future,
- bid and ask greater than zero,
- out-of-the money at the start of the given day.

The two-zero bid rule from the VIX methodology (Exchange 2009) is not considered in our analysis, following the results in Osterrieder et al. (2019), that this, on average, has a negligible effect on the index itself.

This leads to an average of 500 available options per day between January 2018 and February 2018. For the intra-day analysis, we compute the eligible options between 08:30 a.m. (CDT) and 09:00 a.m. (CDT) and keep this set of options constant throughout a given day. The calculations are then starting at 9:01 a.m. (CDT). We split the data into two subsets: the training and validation data sets where the month of January is used for training and the month of February for testing the out-of-sample accuracy of the model.

## VIX highlights

Before we dive into the deep waters of neural networks to predict the VIX, we want to give a more detailed background on the VIX and the characteristics of the underlying options that are used to compute it. In Sect. 5.1, we will analyse the VIX formula and its additive term, the second term of Eq. (1). Then, to motivate our approach of aiming to just use ten options, we show the number of options that are normally needed to predict the VIX in Sect. 5.2. The events on February 5, 2018 are analyzed in Sect. 5.3 to show potential arbitrage opportunities in the VIX market.

### Forward value in the VIX formula

**Fig. 2:**
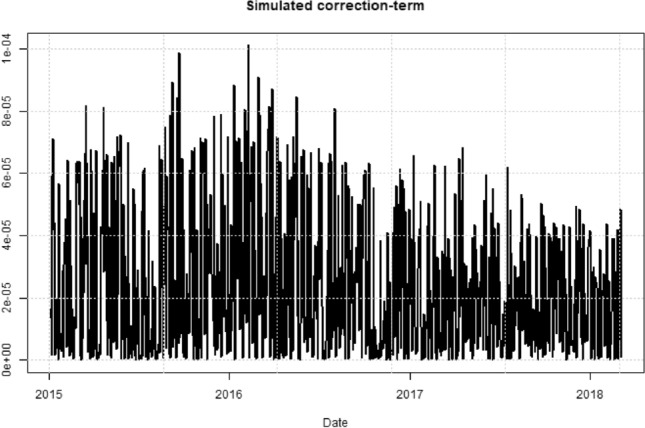
Forward term of the VIX formula

As a way of motivating that a machine learning technique is able to predict the VIX without having knowledge about its underlying theoretical formula, we analysed the second term of Eq. (1). This term is very small compared to the actual VIX, as can be seen in Fig. 2, and can therefore normally be neglected.

### Options in the VIX replication

**Fig. 3:**
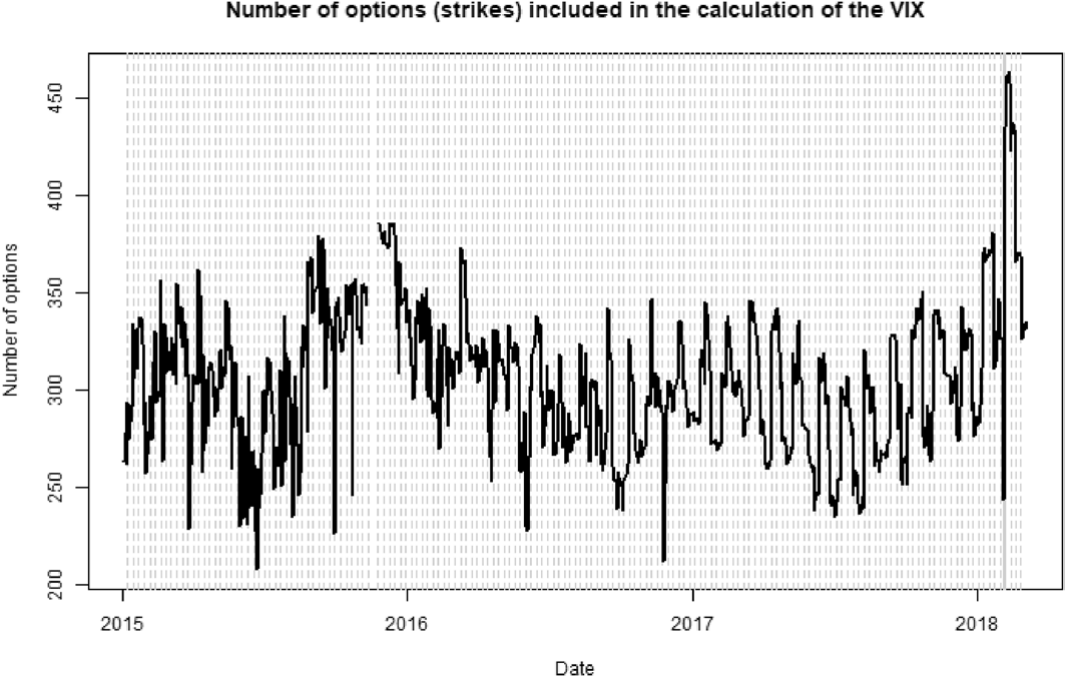
Number of put and call options which are included in our VIX replication

As a preparation for our analysis, we have also replicated the VIX on a daily basis, using the methodology of the VIX white paper (Exchange 2009) to get a better understanding of what is needed to hedge it using options. In Fig. 3 we see the evolution of the number of options for replicating the VIX between 2015 and 2018, which fluctuates between 200 and 450.

**Fig. 4:**
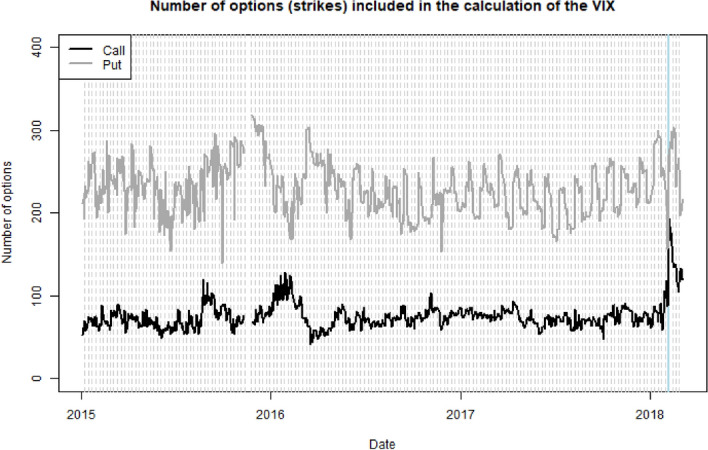
Number of put and call options for the VIX replication, separated by puts and calls

Plotting the previous values separately by puts and calls, we see in Fig. 4 that we need, on average, 100 call and 250 put options for the replication.

### The VIX and February 5, 2018

**Fig. 5:**
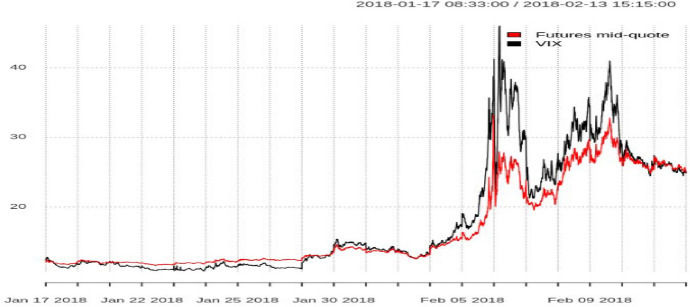
VIX spot and mid quote on February 5, 2018

The events on February 5, 2018, when the VIX moved the most in a single day in the index’s 25-year history, became a strong motivation for our analysis, see Fig. 5. On this day the VIX closed with 37.32 points, an increase of 20.01 points over the previous day, corresponding to an increase of 115% in 1 day. The extraordinary move coincided with a steep sell-off in the equity markets with the S&P 500 index falling by 4.1%. This event shocked the financial world and led to renewed accusations of market manipulation. On that day, we can observe a substantial deviation between the VIX and VIX futures. However, arbitraging away that difference is difficult, due to the sheer number of options that are theoretically needed, to fully replicate the VIX. Our approach later will simplify that task slightly, since we only need ten options to predict the VIX.

## Using an LSTM network for predicting the VIX

We will use an LSTM network which is trained on SPX option quote data to predict the VIX value. For a given volatility surface, different ways of using the option quote data can be envisioned to predict a given volatility surface. Therefore, on purpose, we do not use the VIX formula in our calculation, we simply use option quote data to train the network. The LSTM should be able to rediscover an appropriate way of combining this information, we do not want to impose any restrictions on it. The features we use for our neural network are the log-returns of the mid-quotes of each option. The ultimate goal of the LSTM is find the appropriate weights and transformations of the S&P 500 option prices so that the VIX can be predicted.

In Sect. 6.1 we describe the neural network architecture and in Sect. 6.2 we show the performance of the network for predicting the VIX.

### Neural network architecture

The chosen architecture consists of one LSTM layer with 50 nodes, and one output layer with one node. The initialization is using the Glorot/Xavier uniform initializer, the orthogonal initializer for the recurrent weights and zeros for the bias vector. For the activation function we use tanh. We do not use batch normalization since we only have a batch size of ten. Our data is normalized by computing log-returns of the prices. For regularization, we use a drop-out rate of 0.1. We have decided to use the ADAM optimizer with an initial learning rate of 0.9 for the momentum decay hyperparameter and 0.999 for the scaling decay hyperparameter. The features we use are the log-returns of option prices of out-of-the-money put and call options. At the beginning of every day, we fix the set of options. The idea behind this is to simplify the process of actually trading those options. For the loss function, we are considering both the mean-squared error (MSE) for predicting the VIX returns and the categorical entropy for predicting up and down moves.

### Predicting the VIX

On a normal day, about 350 options are needed for the replication of the VIX, see Fig. 4, consisting of 250 put options and 100 call options. We will train our network on 10, 100, 200 options respectively, equally split between put and call options. Our training set is the intraday data in January 2018, the validation set is the data for February 2018, with a total of 1.68 m and 1.52 m observations, respectively (for 100 options).

**Fig. 6:**
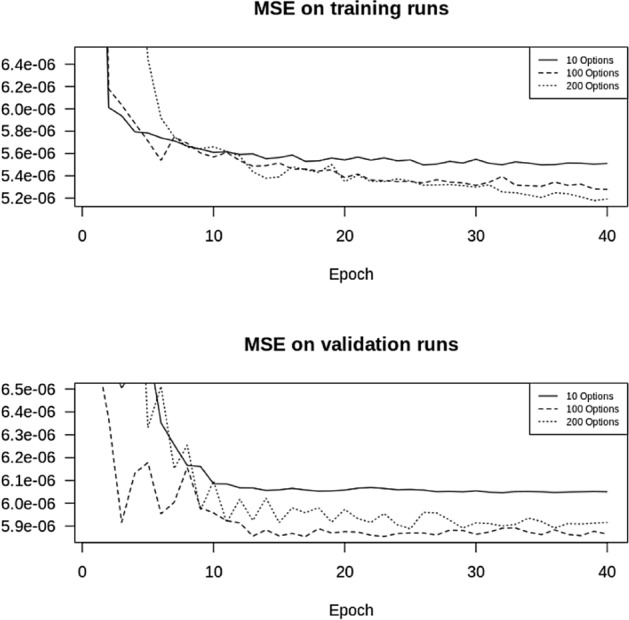
MSE with one layer and 50 LSTM units, with 10, 100, 200 options

In Fig. 6, we show the mean-squared error (MSE) of the forecast as a function of the number of epochs, for 10, 100 and 200 options.

We summarize the MSE in Table 1.

**Table 1:** MSE with a varying number of options

	10 options	100 options	200 options
Training data MSE	5.51e$-$06	5.28e$-$06	5.19e$-$06
Validation data MSE	6.05e$-$06	5.86e$-$06	5.92e$-$06

From Table 1 we conclude that using 100 instead of 10 options to predict the VIX, improves the MSE by about 3%, whereas doubling the number of options to 200 does not improve it anymore. As expected we also see that the MSE on the test set is better than on the validation set. From this we conclude that there is definitely no need to use all 200 options in the prediction, 100 are enough, yet, since the improvement in the MSE is only marginal, ten options are already enough. We have thus found a substantial simplification of the VIX index methodology.

**Fig. 7:**
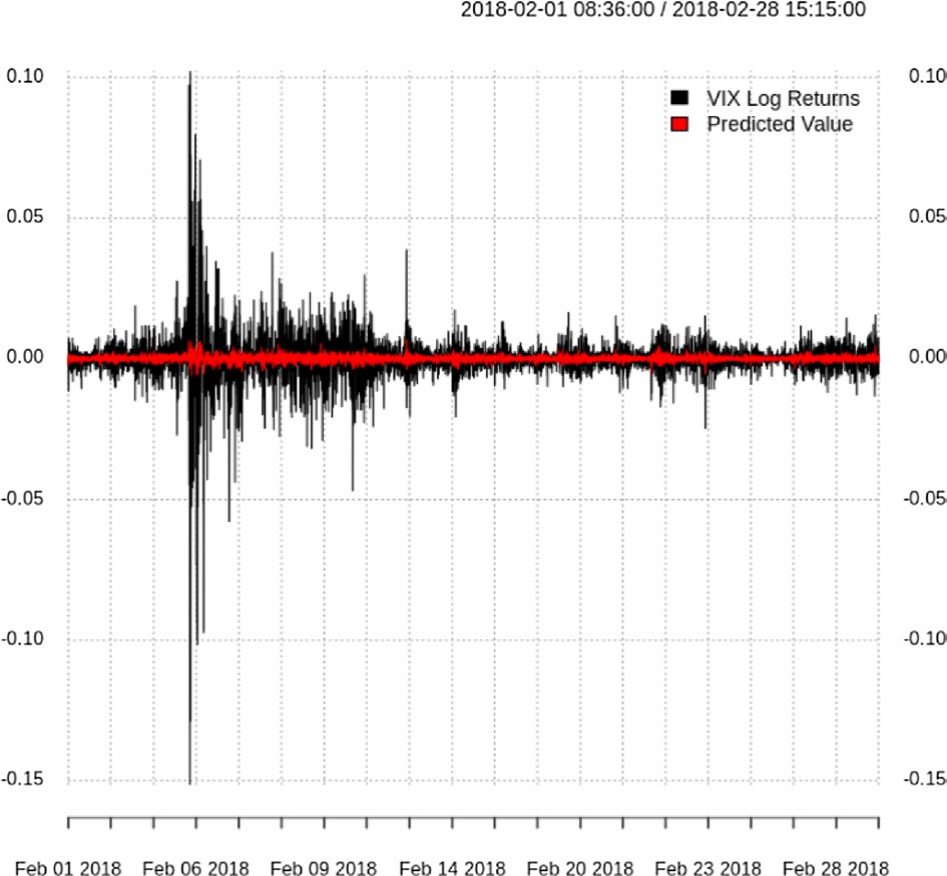
Actual vs. predicted log returns using the validation data set

For further visualization, the predicted VIX spot returns are compared with the actual values in the validation data set for ten options in Fig. 7.

To judge how good the prediction is, we use the naive prediction which consists of just using the current VIX value as our forecast for the next time-step. The MSE for our benchmark is 5.43e$-$05 vs an MSE of 4.08e$-$05 for our prediction.

**Fig. 8:**
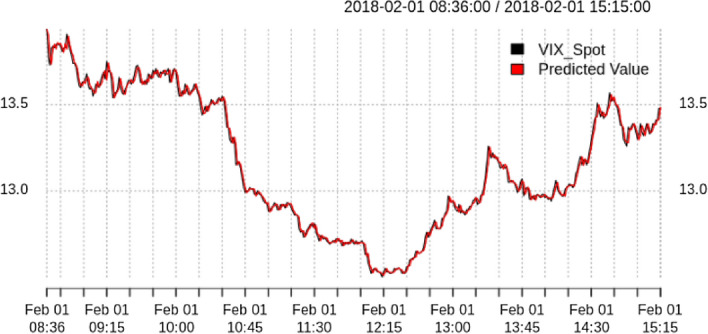
VIX vs. one step ahead prediction

Figure 8 shows the 1 min ahead prediction of the VIX for one specific day, calculated as:

$$\begin{aligned} \hat{p}_i = p_{i-1} exp(\hat{r_i}) \end{aligned}$$

where $\hat{p}_i$ is the predicted price at time *i*, $p_{i-1}$ is the price at time $i-1$ and $\hat{r_i}$ is the predicted return at time *i*.

As you can see, both the predicted and the actual value coincide very well. The MSE of our approach is better than the naive approach, but we need to shed more light on that result. We want to know how often our model predicts the correct direction of the price move. Using the categorical cross-entropy as loss function, with the sign of the option returns as input features and the sign of the VIX returns as output feature, we achieve an accuracy of $61.28\%$ on the out-of-sample data, as can be seen in Table 2. From this, we report the positive predictive value as 60.5% and the negative predictive value as 63.5%, with a sensitivity and specificity of 36% and 82.5% respectively.

**Table 2:** Confusion matrix for our classification approach in February 2018

		Actual signal	
		$-\,$1	1
Predicted signal	$-\,$1	1252	721
	1	2222	3405

PPV	NPV	Sensitivity	Specificity
0.6051	0.6346	0.3604	0.8253

**Fig. 9:**
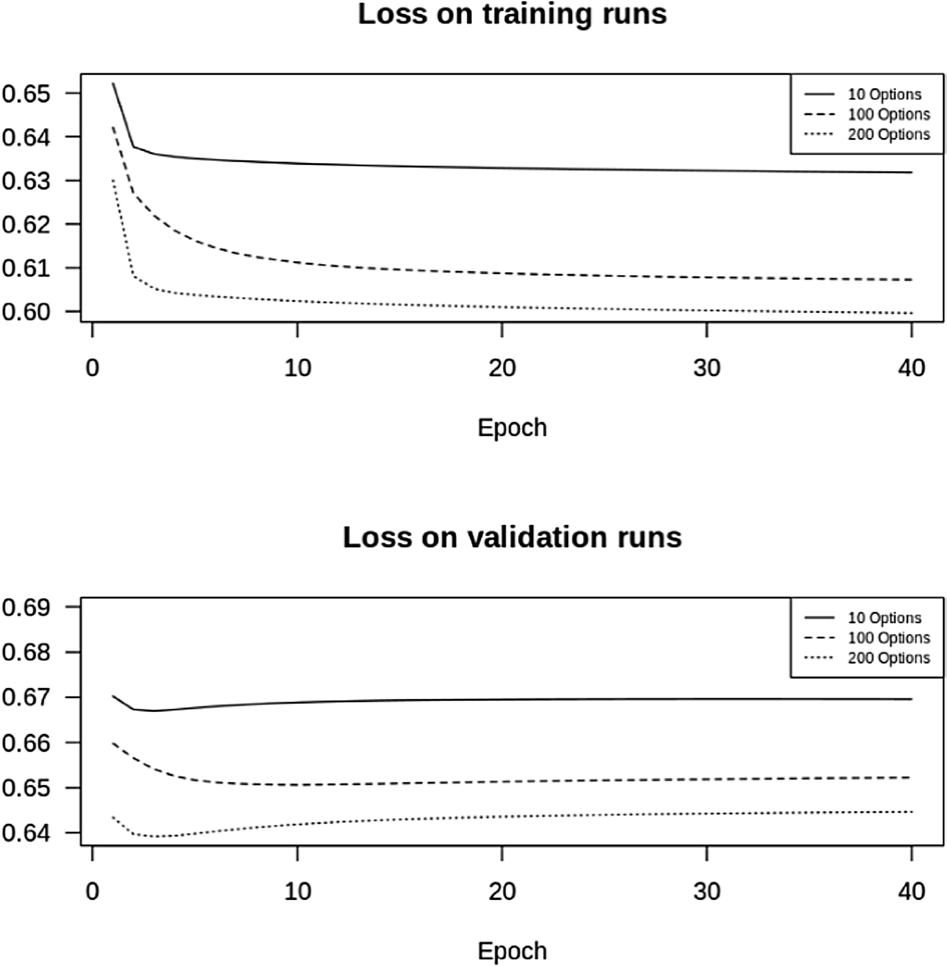
Log Loss with 10, 100 and 200 options for our classification approach in February 2018

Figure 9 shows the improvement in the log loss as a function of the number of epochs. As expected, we get an improvement if we increase the number of options in our calculation. Remember that the VIX white paper (Exchange 2009) mandates that we use all out-of-the money options until we have two consecutive non-zero bids. Here, with only ten options we obtain good results, which makes it substantially easier to actually predict the VIX. Furthermore, the CBOE methodology mandates a time-varying set of options, which can potentially change every 15 s. In our approach, we have at least fixed the universe of available options in the morning and will use those throughout the day.

### Random forests for the VIX

As a comparison to our deep learning approach, we have also used a more traditional machine learning approach, random forests. Our approach is based on Breiman (2001) random forest implementation as described in Breiman (2001). Our random forest consists of 1000 trees, with three variables tried at each split, and input features consisting of the ten most important OTM options. Using the out-of-sample data for February 2018, we have recorded the results in Table 3, with an accuracy of $59.9\%$. Here, both the positive and negative predictive value are 60%, with a sensitivity and specificity of 37% and 79% respectively.

**Table 3:** Confusion matrix using random forests in February 2018

		Actual signal	
		− 1	1
Predicted signal	− 1	1295	861
	1	2179	3264

PPV	NPV	Sensitivity	Specificity
0.5997	0.6006	0.3728	0.7913

## Conclusion and summary

To replicate the VIX using the official CBOE formula, one needs about 350 out-of-the money options at any point in time. It has been shown that ten options (five call and five put options) are sufficient when used as input features for a neural network with one LSTM layer, to predict the VIX with an accuracy of 61.2%, which is slightly larger than using a random forest approach. Large deviations between VIX futures and the VIX arise on an intra-day scale. Using our methodology one might be in a better position to exploit any such arbitrage opportunities than is nowadays possible. Nevertheless, the option market is characterized by high transaction costs and low liquidity, which will still make it challenging to benefit from those differences between the VIX futures and its underlying. Further research in this area needs to focus on four aspects. Our approach, on purpose, was based on a simple LSTM to show the benefits of it, whereas future research can focus on refining the neural network architecture. The second aspect is to more precisely describe and analyse the arbitrage strategy that uses an appropriate subset of the S&P 500 options to predict the VIX. Third, due to the deviations between the VIX and its futures, one can also explore a direct replication of the VIX derivatives. Furthermore, it is worth investigating the relation between the VIX index and its derivatives, most notably VIX futures and VIX options in much more detail. We are also confident that future research can shed light on many claims about possible VIX manipulations that have been brought up by market participants over the last few years, most notably unusual trading patterns, which were observed on the market, on the 5th of February and 18th of April, 2018.

## Footnotes

1 If this Friday is a holiday, options will expire 1 day earlier and those are then used during the SOQ.

2 A day contains 1440 min which is 24 h.

3 https://datashop.cboe.com/.
